# The role of Interleukin-1 receptor antagonist as a treatment option in calcium pyrophosphate crystal deposition disease

**DOI:** 10.1007/s11033-021-06457-z

**Published:** 2021-06-01

**Authors:** Alberto Altomare, Addolorata Corrado, Nicola Maruotti, Daniela Cici, Francesco Paolo Cantatore

**Affiliations:** Rheumatology Clinic “Mario Carrozzo”, Department of Medical and Surgical Sciences, University of Foggia, “Policlinico Riuniti” University Hospital, Viale Pinto, 1, 71121 Foggia, Italy

**Keywords:** CPPD, IL-1, Anakinra, Pseudogout, Arthritis

## Abstract

Calcium Pyrophosphate Crystal Deposition (CPPD) disease is characterized by the deposition of calcium pyrophosphate crystals in the cartilage. In most cases, it can manifest as a subclinical condition named chondrocalcinosis, often revealed by joint x-ray examination. In other cases, deposition can cause flares of arthritis, known as acute CPP crystal arthritis. In the last few years, many pathogenic pathways have been discovered. Interleukin-1 (IL-1) plays a key role in the pathogenesis of CPPD disease, both as a mediator of inflammatory response to crystals and as a promoter of damage to articular cartilage. In this review, we investigated the role of IL-1R inhibitor, such as Anakinra, as an alternative to the various therapeutic strategies for CPPD disease, especially among patients resistant to traditional treatment with NSAIDs, corticosteroids and colchicine.

## Introduction

Calcium Pyrophosphate Crystal Deposition (CPPD) Disease is characterized by the deposition of Calcium Pyrophosphate crystals in the cartilage [1]. Fibrocartilage is most commonly involved, especially the menisci and the triangular fibrocartilage complex, but hyaline cartilage may also be calcified [2].

Aging is known as the most significant risk factor for chondrocalcinosis, with an increasing prevalence in elderly people [3, 4]. In fact, it is less than 4% in those under the age of 70 and rises to 27% in those over 85. Several studies show that the average age of presentation is between the seventh and the eighth decade of life [5]. Other risk factors strongly associated with CPPD are hyperparathyroidism[6], hypomagnesemia (alone or as a manifestation of Gitelman’s Syndrome), hemochromatosis (especially in those under 65) and hypophosphatasia, although the role of the latter is debated[7–9].

The most affected joint is the knee. In particular, knee chondrocalcinosis appears to be strongly associated with the presence of previous surgeries [10]. The second most affected joint is pubic symphysis with 33.1% of cases, followed by coxofemoral, with 3.5%, and sacroiliac joint (SI) [11]. According to Abhishek et al., 42% of patients had no knee involvement. Moreover, wrist and hip joints are more commonly affected than symphysis, hip, SI and metacarpo-phalangeal joints. This suggests that radiographs of knees, hips and hands should be performed to adequately screen for chondrocalcinosis [12]. The acromioclavicular joint may be also involved with a prevalence of 1.1% [13].

According to EULAR 2011 guidelines [14] it is possible to distinguish four different clinical presentations of the disease: Chondrocalcinosis, the asymptomatic X-ray finding of crystal deposits in the cartilage, with no joint inflammatory symptoms [15]; Osteoarthritis with CPPD, which appears in joints usually spared by primary osteoarthritis [16, 17]; Acute CPPD crystal arthritis, characterized by acute flare similar to gout and therefore known as Pseudogout. In this form, one or more joints could be affected, with acute inflammation that develops quickly and reaches its peak on the third or fourth day. The inflammatory joint involvement may be accompanied by fever, anorexia and loss of weight. Besides, there will be a raising of erythrocyte sedimentation rate (ESR), α-globulin and C reactive protein (CRP)[18]. Acute attacks most commonly affect the knee, followed by wrist, ankle and hand. Polyarticular involvement is also possible [10]. Subsequent episodes follow the first one at irregular intervals.

Chronic CPPD arthritis is characterized by chronic oligoarthritis or polyarthritis with inflammatory symptoms and signs and occasional systemic upset (with elevation of CRP and ESR) [14]. The knee is the joint most affected by chronic disease too [10], followed by the wrist, with the triangular fibrocartilage calcification, the metacarpophalangeal joints [19], the hip, the glenohumeral joint, the spine [20, 21] and the temporomandibular joint [22].

Diagnosis is based on conventional radiograph (CR) that has a sensitivity of 0.39–0.47 and specificity of 0.95–1 [23–25] and US imaging characterized by high sensitivity 0.85- 0.89 and specificity 0.87–0.9 [25, 26]. CR shows the typical calcification associated with other features such as joint space narrowing, subchondral bone sclerosis, bone cysts and marked osteophytes [10]. It’s to be noted that the absence of calcification on CR does not exclude the diagnosis [27]. US imaging detects the presence of pouring, synovial hypertrophy, and the presence of crystals, although CPP deposits differ depending on the structure observed. In fact, in fibrocartilage, hyaline cartilage and synovial fluid we have hyperechoic deposits without posterior shadowing, while tendon deposits appear like multiple linear hyperechoic structures [28]. Despite a little lower specificity, US has higher sensitivity and it is a non-invasive technique, therefore US imaging should be preferred to CR [25, 27].

The diagnosis of certainty is made with the analysis of the synovial fluid and the direct observation of the CPP crystals. Despite its 100% specificity, this method has a 70% sensitivity, thus one third of the patients could be misdiagnosed [27]. However, crystals observed in the synovial liquid by polarized light microscopy appear in a rhomboid shape with faint birefringence or lack of it [1, 29].

## The role of IL-1 in the pathogenesis of CPPD disease

CPP Crystals activate monocytes and macrophages, but also synoviocytes and endothelial cells [30]. This is due to three main mechanisms. The first one is the recognition of crystals by the Toll-Like Receptors system (TLRs), mainly TLR-2 and TLR-4 in chondrocytes [31]. A second mechanism can account for the formation of a protein envelope on the crystal's surface, including C1q, C5, C6, IgM and IgG that lead to the opsonization of crystals. The third mechanism witnesses an interaction between the crystals and the cell’s membrane, which leads to an intracellular signaling mediated by Syk protein, a tyrosine kinase. This could be a mediator for the internalization of crystals or the cellular response to crystals binding [30].

Interleukin 1 (IL-1) is a cytokine involved in the host response to infection and inflammation. Besides, it has a catabolic and proinflammatory activity, causing the activation of neutrophils and endothelial cells. Three isoforms are known: IL-1α, IL-1β and IL-1RA. IL-1RA is the natural inhibitor of IL-1. IL-1 has a receptor family which includes three members: IL-1RI, IL-1RII and IL-1 accessory protein (IL-1RacP). The intracellular signalling pathway is activated only by the binding between IL-1 and IL-1RI. IL-1β has the lowest affinity for IL-1RI, while it has a higher affinity for IL-1RII, with a nearly irreversible binding. On the contrary, IL-1RA has the highest affinity for IL-1RI and its binding is nearly irreversible. These mechanisms constitute a natural inhibition for the IL-1β signalling pathway [32]. CPP crystals would act on macrophages and monocytes to stimulate IL-1 secretion and sensitize chondrocytes to IL-1 stimulation [33, 34]. The way in which crystals can activate IL-1 production is still debated, but it seems that crystals interact directly with NALP3, ASC and caspase-1, components of the inflammasome. Thus, caspase-1 catalyzes the cleavage of pro-IL-1β, inducing the maturation of IL-1β [30, 35–37]. To date, it has been shown that CPP crystals induce a down regulation of IL-1Ra, the natural antagonist of IL-1, leading to an increasing activity of IL-1 [38]. Higher levels of IL-1β induce an increasing production of downstream cytokines such as PGE_2_ and IL-8 in osteoblasts, with an abnormal local bone resorption and a reduced new bone formation [39], and TNF-α, IL-6, CXCL-1 and CXCL-8, with proinflammatory effects [30]. PGE_2_ production is stimulated by IL-1β at many levels. In fact, IL-1β is able to induce soluble phospholipase A2 (sPLA2), which determines the release of arachidonic acid from membrane phospholipids. Arachidonic acid is converted to prostaglandine G (PGG) by cyclooxygenase 2 (COX-2), which is induced by IL-1β synergistically with TNFα [40]. Thus, PGG is converted into PGH2 by sPLA2 and this is finally converted into PGE2 through cytosolic PGE synthase (cPGES) and mitochondrial PGE synthase (mPGES). The latter has two isoforms: mPGES1 and mPGES2; the first is induced by IL-1β [41].

A further role of IL-1 arises from the observation that it is able to inhibit peroxisome proliferator-activated receptor γ (PPARγ) expression, both in human and rat chondrocytes [42]. Experimental models have shown that ligands of PPARγ, such as 15-deoxy-prostaglandin-J2 (15dPGJ2) and leukotriene B4 may bind PPARγ [43] with antagonist effects to IL-1. In fact, it has been shown that, through its binding to PPARγ, 15dPGJ2 is able to inhibit IL-1-dependent COX-2 induction, NO production and metalloproteinase 1 (MMP-1) and metalloproteinase 13 (MMP-13) activation in human fibroblast [44–46]. Although 15dPGJ2 is not produced in mammalian cells, it is reasonable that other PPARγ ligands may give the same effects.

The role of IL-1 is even supported by the evidence that colchicine, used in the prevention of new acute attacks, is able to suppress maturation and release of IL-1β [35]. As shown on a model of gouty arthritis rat, suppression of IL-1β gives a reduction of downstream signalling cytokines such as IL-6, MCP-1, KC with an improvement of hyperalgesia and inflammation [47]. Some authors suggested that IL-1 could have an autocrine activity mediated by myeloid differentiation factor 88 (MyD88), which activates I_κ_B kinases (IKKs). This in turn phosphorylates I_κ_B, removing its inhibition on NF-Kβ, that stimulates the nitric oxide (NO) production, amplifying the proinflammatory effect [31, 48]. In fact, NO production has a synergistic effect to induce cartilage degradation, giving an inhibition of type II collagen and aggrecan production and an enhancement of MMPs. Moreover, NO is able to inhibit IL-1Ra production and gives a higher susceptibility to injury and pro-oxidant damage to chondrocytes [32].

Moreover, IL-1β induces MMP-1 and MMP-13 expression, determining collagenase type II degradation, which is responsible for the cartilage damage, mostly in inflammatory arthritis [32]. The articular damage is also induced by the inhibition of type II collagen and proteoglycans production. Cartilage damage is also favored by the ability of IL-1 to inhibit the tissue inhibitors of matrix metalloproteinases (TIMPs), which, in normal conditions, determines the inhibition of MMPs. To be noted: it has been shown that TIMP-1 activity was inhibited in IL-1β-stimulated chondrocytes [32].

Finally, IL-1β has a role on aggrecans, other components of the articular cartilage that confer compressibility to the cartilage. They bind hyaluronan and link protein to form a macromolecular complex which interposes itself within collagen molecules. The loss of aggrecan is considered a critical event in the cartilage destruction process and in its subsequent collagen degradation [49, 50]. Aggrecans are degraded by a family of proteases or aggrecanases named ADAMTS, which involve 19 gene products. It has been shown that IL-1β is able to induce mostly ADAMTS-9 with loss of aggrecans and cartilage damage [51].

As shown by these studies, the final effect of IL-1 is the stimulation of local and systemic inflammation, promoting the recruitment of inflammatory cells at the site of inflammation, inducing the expression of adhesion molecules on the surface of endothelial cells and attracting chemokines from stromal cells, with final cartilage damage.

## Management of CPPD disease and role of IL-1 antagonist as treatment option

According to the EULAR recommendations of 2011, in case of asymptomatic chondrocalcinosis no treatment is necessary. In acute CPP crystal arthritis, application of icy compresses and rest of the joint are useful, although their effectiveness is not proven by scientific evidence and their use comes from clinical observation. Other options are aspiration of synovial fluid and injection of glucocorticoids in the intra articular setting, whether it is monoarthritis or oligoarthritis [14].

In case of polyarthritic acute attacks, oral NSAIDs and colchicine at the dose of 0.5 mg three or four times a day may be useful, with or without a 1 mg load dose. In administering these drugs, possible side effects should be considered. For colchicine, the most frequent side effect is diarrhea, while for NSAIDs there are many and well-known side effects, including gastrointestinal bleeding, cardiovascular events and effects on the kidney.

Both oral administration and IV administration of glucocorticoids (GCS) may be effective in acute CPP arthritis. Moreover, low doses of GCS may be also used in chronic CPP arthritis, whereas NSAIDs and colchicine are ineffective. For the purposes of prophylaxis of new attacks, studies suggest the use of colchicine at the dose of 0.5–1 mg/die or low doses of NSAIDs [14].

As stated by Parperis et Al. in their systematic review, although NSAIDs, colchicine and GCS are widely used in the treatment of CPP arthritis, there are no well-designed studies that assess their effectiveness and the treatment for CPPD disease is mostly based on gout treatment [52].

Anakinra, an IL-1R inhibitor, may be an option for patients who can’t be administered NSAIDs, such as elderly patients with kidney failure or patients resistant to treatment with NSAIDs, corticosteroids and colchicine. The rationale in the use of this drug, already used to treat rheumatoid arthritis, would be to inhibit the interaction between IL-1 and its receptor. In fact, anakinra inhibits the binding between IL-1α/IL-1β and IL-RI and it has been approved for the treatment of rheumatoid arthritis, Still’s disease, cryopyrin associated periodic syndrome (CAPS) and familial mediterranean fever [53]. In consideration of his short half-life it is well tolerated and may thus be considered a further therapeutic strategy when NSAIDs and colchicine can’t be used [54]. A first case report of the use of anakinra on steroid-resistant chondrocalcinosis was related by McGonagle et al. A 63-year-old patient with a three months history of acute CPP crystal rthritis with swelling, pain and erythema of the right first finger, was given anakinra at the dosage of 100 mg per day by subcutaneous injections (as described by So et al. in a case series of ten patients with gouty arthritis [55]), with an improvement in 14 days. After 3 months the patient was asymptomatic and he was able to stop indomethacin and prednisolone, with normal CRP and ESR levels [56]. Subsequently, other case reports have been reported on the efficacy of anakinra in chondrocalcinosis resistant to NSAIDs, methotrexate and steroids [57, 58]. Among these, Moltò et al. reported a small case series of five patients with CPP-induced arthritis, with a flare lasting, on average, 45 ± 15 days. Resistant to standard treatments, they received 100 mg/die anakinra for three days. Four out of five patients showed rapid clinical response with flare recovery after an average of three days, associated with reduction of pain and CRP levels. Two patients didn’t need any maintenance regimens or retreatments for one year, the other two patients for 6 months [59] (Table 1).

**Table 1:** Studies dealing with use of anakinra in CPP crystals arthritis

Studies	Type of study	Number of patients	Regimens	Effectiveness	Safety
Successful treatment of resistant pseudogout with anakinra [56]	Case report	1	100 mg/day sc for 1 year	Asymptomatic within 3 months with CRP and ESR levels normalized	No adverse events were reported
Efficacy of anakinra in articular chondrocalcinosis: report of three cases [57]	Case report	3	Case 1. 100 mg/day sc + prednisone + MTX for 1 year Case 2 and 3. 100 mg/day sc for 3 months	Case 1. no further flares with synovitis in 3 joints (7 at baseline) Case 2 and 3. no response and stopped after 3 months	No adverse events were reported No adverse events were reported
Interleukin 1A blockade improves signs and symptoms of chronic calcium pyrophosphate crystal arthritis resistant to treatment [58]	Case report	1	100 mg/day sc for 8 months	Knee arthritis remission with CRP and ESR within 1 week	No adverse events were reported
Efficacy of anakinra for refractory acute calcium pyrophosphate crystal arthritis [59]	Observational retrospective study	5	100 mg/day sc for 3 days	4 patients had joint pain reduction with ESR and CRP levels reduction in 3 days. 1 patient had no response	Local skin reaction at the injection site in 1 patient
Efficacy of anakinra in calcium pyrophosphate crystal-induced arthritis: a report of 16 cases and review of the literature [54]	Observational retrospective study	16	75% had 100 mg/day sc for 3 days in 12 patients 25% had 100 mg/day sc for 7 days-6 months	Among 12 patients treated for 3 days 67% had good response, 25% had partial response, 8% had no response Among the other 4 patients, 2 had good response, 1 had partial response and 1 had no response	No adverse events were reported
Use of anakinra in hospitalized patients with crystal-associated arthritis [60]	Observational retrospective study	100 patients with gout and CPPD disease with 115 episodes of arthritis	80.8% of episodes were treated with 100 mg/day sc for 1–3 days 19.2% of episodes were treated with 100 mg/day sc for more than 3 days	73% of episodes had complete or partial response within 4 days after the first dose 57,3% of episodes had complete or partial response within 1 day after the first dose 6% of episodes had partial response… 5.2% of episodes had no response……… ………	No adverse events were reported. In 29 patients with concomitant infection there were no signs of worsening
Biologics in the treatment of calcium pyrophosphate deposition disease: systematic literature review [61]	Systematic literature review	76	74 patients (97.7%) received anakinra at dose of 100 mg/day Among these patients, 76.1% was treated with 1–3 days, 23.9% was treated for 5–9 days	80.6% had clinical response among those patients with acute CPP crystals arthritis 42.9% had clinical response among those patients with chronic CPP crystals arthritis	Adverse events were reported in 4.1% of patients. Mainly skin reaction at site injection and respiratory infection

Similar results are shown by Ottaviani et al. In their case series, 16 patients with CCP induced arthritis, with a mean age of 80.2 years and mean duration of flare of 10.7 days, showed at baseline mean VAS pain of 78.7, mean tender joint count (TJC) of 6.9 and mean swollen joint count (SJC) of 6.3. CRP levels were evaluated with a mean of 109.8 mg/L at baseline. All these patients were resistant to conventional treatment (corticosteroids, NSAIDs and colchicine) and 100 mg/day anakinra was administered for 3 days to 12 patients. The other 4 patients were administered anakinra at the same dosage but for 7 days, 8 days, 1 month and 6 months, respectively. The authors observed that, among the 12 patients treated with the So et al. protocol, 67% showed a good response, 25% a partial response and 8% had no response. Interestingly there was a decrease of mean VAS pain to 28.8, mean TJC to 2.0, SJC to 1.9 and CRP levels to 21.1 mg/L. It’s to be noted that at baseline joint ultrasonography (US) was performed on all the symptomatic joints of 14 patients, showing effusion and/or hypervascularization. After treatment with anakinra, joint US was performed on day 4 to 12 patients, showing the complete resolution of synovial hypervascularization signal. A relapse of the disease was noted after a mean period of 7.8 months [54].

Thus, the role of anakinra in the crystal induced arthritis was endorsed by Liew and Gardner. They conducted an observational retrospective study on 100 patients with 115 episodes of gout and acute CPP crystal arthritis, treated with anakinra at various dosages (100 mg/day for two days, 100 mg/day for three days or more). 84 episodes had a partial or complete response within four days from the first dose and 66 episodes had partial or complete resolution after one day of treatment. There were 7 episodes of partial response and 6 episodes with no response. Moreover, in this study anakinra showed a good safety profile. As a matter of fact, it was administered to 29 patients with concomitant infections (localized and systemic) treated with antibiotic therapy. No patients showed signs of worsening of the infection [60].

More recently, a systematic literature review by Cipolletta et al. showed that on 67 patients with acute CPP-crystal arthritis, the 76.1% was treated for 1–3 days, whereas the 23.9% required 5–9 days of anakinra administration for symptom remission. This suggests that longer treatment may be necessary in patients with a longer duration of the disease or with chronic CPPD disease. However, the most important effect was observed on acute CPP crystal arthritis with a significant reduction of TJC, SJC, VAS pain and CRP level [61].

As for relapse chances after anakinra treatment, Parperis et al. reported two studies where the relapse of acute CPP-crystal arthritis after anakinra administration of was 6/16 and 9/33 patients, although another study showed no signs of relapse on 5 patients after a 6 and 12 months update [52].

Recently, Dumusc et al. performed a randomized controlled double-blinded trial on 15 patients to evaluate the efficacy of a three-day course of anakinra versus prednisone to treat acute CPP arthritis. The authors reported that anakinra and prednisone have similar effectiveness in acute CPP arthritis. Anakinra seems to have a faster onset of action than prednisone. Thus, the authors suggested that anakinra may be useful in patients with comorbidities to shorten their hospitalization [62].

As shown by Cipolletta et al. clinical response to anakinra was more evident in acute CPP crystal arthritis, with a response rate of 80.6%, than in chronic CPP crystal arthritis (response rate of 42.9%), although the last case probably depends on the small sample size, which doesn’t allow to draw any definitive conclusion [61]. However, all the studies reported show that in chronic CPP crystal arthritis, anakinra could inhibit onset of a new flare of disease.

Interestingly, in all these studies, anakinra has shown to be relatively safe, with no or poor adverse effects, mainly cutaneous reaction on the injection site.

## Conclusions

In this review we have highlighted the pivotal role of IL-1 in the pathogenesis of the often misdiagnosed CPPD disease and how this cytokine has a role in the events that lead to inflammatory modifications and joint damage. IL-1 is produced by activated macrophages and monocytes (stimulated by CPP crystal shedding in joint space); it can induce and orchestrate a proinflammatory response resulting from the activation of various enzymes that mediate the production of further proinflammatory cytokines, such as PGE2, or proinflammatory mediators like NO. This response is synergized by the suppression of other factors that have an antagonist effect compared to IL-1, such as PPARγ. Another important role of IL-1, as seen before, is the ability to induce articular damage through the activation of proteinases such as MMP-1, MMP-13 and ADAMTS-9 and to suppress their inhibitors, like TIMP-1, with the result of type II collagen and aggrecan degradation. All these actions have the final effect of inducing an inflammatory response into the joint with the formation of an inflammatory infiltrate. It determines the clinical manifestations of the disease that may be an acute arthritis attack or a chronic arthritis with articular damage often resembling what we can find in osteoarthritis. The disease can more often still be asymptomatic.

Given the role of IL-1 in the pathogenesis of CPPD disease, many authors tried to administer drugs that act on this mechanism. The first used for this aim was anakinra. As reported in this review, it has shown encouraging results, although there are no randomized controlled trials to confirm its efficacy in the treatment of CPPD disease. All the data on the effectiveness derive from various case series, although these results show that anakinra may be a useful tool in the treatment of the disease, most of all in patients that are resistant to other treatment options such as NSAIDs, corticosteroids and colchicine.

Moreover, anakinra seems to be a good alternative in relation to safety too, since the data from the reported case-series have not shown serious adverse effects and, when present, they have been mainly attributable to skin reactions on the injection site.

Therefore, IL-1 R blocking therapy could be taken into account in patients with acute and chronic CPP crystal arthritis with comorbidities, to reduce hospitalization times, or in patients where NSAIDs, colchicine and GCS are ineffective or contraindicated. Given the relative safety of anakinra, there would not be absolute contraindications, although there aren’t any studies that investigated this aspect. Thus, the principal contraindication could be the neutropenia, that may be observed in course of treatment or concomitant infection.

Given the good results shown by anakinra in the treatment of CPPD disease and other crystal-induced arthritis, like gout, other studies should be carried out to demonstrate its efficacy, to eventually find a place among the possible therapeutic strategies in CPPD patients.
